# Localized surface plasmon resonances dominated giant lateral photovoltaic effect observed in ZnO/Ag/Si nanostructure

**DOI:** 10.1038/srep22906

**Published:** 2016-03-11

**Authors:** Ke Zhang, Hui Wang, Zhikai Gan, Peiqi Zhou, Chunlian Mei, Xu Huang, Yuxing Xia

**Affiliations:** 1 The State Key Laboratory of Advanced Optical Communication Systems and Networks, Department of Physics and Astronomy, and the Key Laboratory of Thin Film and Nano-microfabrication Technology of the Ministry of Education, Shanghai JiaoTong University, 800 Dongchuan Rd, Shanghai 200240, P.R. China

## Abstract

We report substantially enlarged lateral photovoltaic effect (LPE) in the ZnO/Ag/Si nanostructures. The maximum LPE sensitivity (55.05 mv/mm) obtained in this structure is about seven times larger than that observed in the control sample (7.88 mv/mm) of ZnO/Si. We attribute this phenomenon to the strong localized surface plasmon resonances (LSPRs) induced by nano Ag semicontinuous films. Quite different from the traditional LPE in PN junction type structures, in which light-generated carriers contributed to LPE merely depends on direct excitation of light in semiconductor, this work firstly demonstrates that, by introducing a super thin metal Ag in the interface between two different kinds of semiconductors, the nanoscale Ag embedded in the interface will produce strong resonance of localized field, causing extra intraband excitation, interband excitation and an enhanced direct excitation. As a consequence, these LSPRs dominated contributions harvest much more carriers, giving rise to a greatly enhanced LPE. In particular, this LSPRs-driven mechanism constitutes a sharp contrast to the traditional LPE operation mechanism. This work suggests a brand new LSPRs approach for tailoring LPE-based devices and also opens avenues of research within current photoelectric sensors area.

The lateral photovoltaic effect (LPE) has been widely employed in optical transducers and sensors especially for those applications where precise autocontrol measurement is necessary such as machine tool alignment, surface profiling, rotation monitoring and robotic vision owing to its linear relation between the instant electrical signal output and the laser spot position illuminated on the hybrid structure surface. On this account, series of jobs recently have been carried out aiming to seek much more reasonable systems which could present with better sensitivity and higher linearity such as hydrogenated amorphous silicon^1^,^2^,^3^, quantum dots^4^, and the blended perovskite based heterojunctions^5^ since it was first discovered by Schottky^6^ and then expanded by Wallmark in floating Ge p-n junctions in 1957^7^. To date, stemming from this desire, our group also have proposed a variety of nano metal (metal-oxide)-semiconductor (MS/MOS) type or MOS-related structures and have achieved a great deal of remarkable photoelectric effect, including large LPE^4^,^8^,^9^,^10^,^11^ and laser-induced bipolar resistance effect^12^,^13^.

Recently, researches of the nanoscale Ag coupled metal-dielectric hybrid systems have drawn more and more attentions due to the the metallic particles and their clusters induced surface plasmon resonance which has proved to be tight dependent on the shape and size of metal nanostructures^14^,^15^. These peculiar nanostructures are found to exhibit intensely nonlinear optical phenomenon and distinctively electronic transport properties^16^ because of the distinguished localization and enhancement of the electric and magnetic fields in the visible and infrared spectral ranges, and thus provoking vital important potential in the flourishing applications such as optical spectroscopy, sensors, solar cells, light emitting diodes (LED) and so on^17^,^18^,^19^,^20^,^21^,^22^,^23^.

Hence on this occasion, we put forward a novel blending nanostructure ZnO/Ag/Si (substrate) where the intermediate Ag semicontinuous film can trigger abundant localized plasmon excitations (hot spots) and arouse strong resonance of the local field with the incident optical field near the metal-dielectric interfaces. As a result, it is found to effectively provide large amount of high energy conducting electrons and thus leading to a major leap for the output of LPE.

## Results and Discussion

All the experiments were performed in the ZnO/Ag/Si and ZnO/Si nanostructures. The thickness of the N-type Si (111) wafer is around 0.3 mm and the resistivity is in the range of 50–80 Ω cm at room temperature. The Ag nanoscale films were deposited by dc magnetron sputtering at room temperature, and the nominal thickness controlled by different grown time was ranging from 2.7 nm to 13.5 nm. The ZnO layer was then fabricated by magnetron reactive sputtering under the deposition pressure 0.6 Pa for Ar and 0.15Pa for O_2_, and the identical thickness was approximately 28.5 nm. The samples were labeled as sample 1 to sample 4 in accordance with the sequential silver thickness 2.7 nm, 8.1 nm, 10.8 nm and 13.5 nm. Here the no silver intermingled ZnO/Si was regarded as the control sample. As a matter of fact, the deposition rate of Ag and ZnO determined by the stylus profile meter on thick calibration samples were checked out to be 1.35 Å/s and 0.95 Å/s respectively. All the samples were scanned spatially with a He-Ne laser focused on a roughly 50 μm diameter spot at the surface and without any spurious illumination (e.g. background light) reaching to the samples. Furthermore, all the contacts (less than 1 mm in diameter) to the film were formed by alloying indium and showed no measurable rectifying effect. Other experimental details were similar with our recently published papers on LPE^12^,^13^.

**Figure 1:**
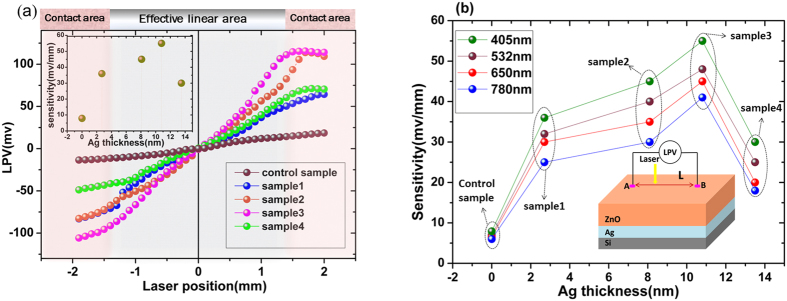
(**a**) Specific LPVs from the control sample to sample 4 as a function of laser position with the 405 nm incident laser. The inset shows different sensitivities vs various Ag thickness (Sensitivity here refers to the differential value of the LPV output over the displacement of laser spot). The shadow regions illustrated with gray and reddish color represented the effective linear area and the contact area respectively. (**b**) Ag thickness-dependence of LPV sensitivities with different laser wavelength (here laser power used is unified as 4 mw). The inset is the schematic diagram of LPV measurement setup. The distance of the two contacts in this letter is identically set up to be 4 mm.

As shown in the Fig. 1a, when the laser spot is impinging on the ZnO side of nanostructures (see the inset of Fig. 1b), all the values of measured LPVs in these systems basically have presented with linear change trend versus the laser spot position as we have reported before^9^. The nonlinearity (spatial resolution for 100 μm) of the effective linear area (namely the distance between the innermost side of the two contacts) was kept in a range of 3–10% in this report. Clearly, these results were much smaller than the lowest nonlinearity value 15% required in the practical application, suggesting an appropriate candidate for the PSD devices. Besides, all the LPVs output induced by the 405 nm laser have obtained their maximum values respectively when the incident light is closest to the contacts (namely, the electrodes), and then as the spot scanned away from the indium electrodes, the values displayed a fitted linear decrease and dropped to zero at the midpoint. Yet, here the unexpected fact is that the largest sensitivity signal 55.05 mv/mm detected in sample 3 (see the pink dot depicted in the inset of Fig. 1a) was about seven times larger than the one 7.88 mv/mm tested in the control sample ZnO/Si.

To better investigate this particular phenomenon, we also made a series of additional LPV tests for all these samples with the varying laser wavelength from 405 nm to 780 nm within the 4 mm contacts spacing as shown in Fig 1b. Indeed, we can discover that all the samples embedded with the semicontinuous Ag film have unambiguously exhibited better sensitivities compared with the control sample ZnO/Si in the visible and near infrared region. Therefore, it is clear that the Ag semicontinuous film in these complex nanostructures is certain to play a crucial role in modulating the optical and electrical properties of the multilayer systems.

It has been well known that the silver embedded nanoscale metal-dielectric composite materials can generate strong localized surface plasmon resonances (LSPRs) at frequencies in the visible range since its dielectric constant has a large negative real part and a relatively small imaginary part^21^,^24^. Given this peculiar behavior, many positive results have been achieved in the Ag nanostructure enhanced photoluminescence (PL) material researches^18^,^25^, especially for the band gap emission (~370 nm) of ZnO hybrid systems in recent years because that the metallic LSPRs here can not only take the special responsibility for the more efficient absorption of excitation light but also assist in radiating the consequent fluorescence emission of nearby molecules to the far-field^26^. Therefore, PL spectra are always clues to the presence of potential LSPRs.

Based on this fact and considering that the silver semicontinuous interlayer film in this research was also processed in nanoscale, we performed a detailed investigation of the PL spectrum to check whether these samples show some LSPRs characteristics as an attempt.

**Figure 2:**
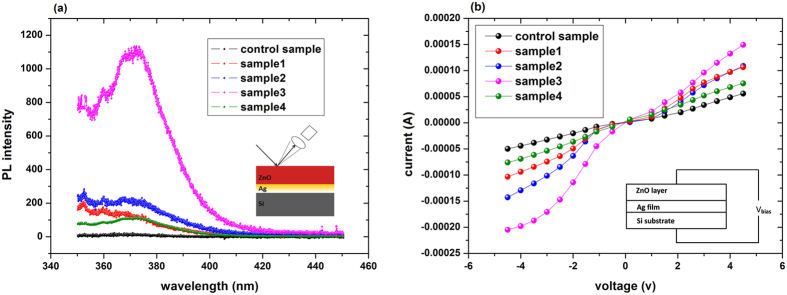
(**a**) Photoluminescence spectra of the silver semicontinuous film mediated ZnO hybrid nanostructures and the control sample ZnO/Si fabricated on N-type Si substrate. The inset shows the schematic drawing of the PL measurement configuration. (**b**) I-V curves of the nanostructures with different Ag thickness, the inset is the schematic circuit of the sample measurement.

The Fig. 2a shows the PL spectra of all the five different samples. We find the sample 3 (the pink line) recorded the strongest PL peak. This indicates that the nanoscale (10.8 nm) Ag granular films in the interface of sample 3 can cause the strongest LSPRs.

Likewise, the sample 1, sample 2 and sample 4 showed relatively weak PL peak due to relatively weak LSPRs. The control sample presented with a practically negligible PL peak because there is no Ag in the interface to stimulate the metallic LSPRs.

**Figure 3:**
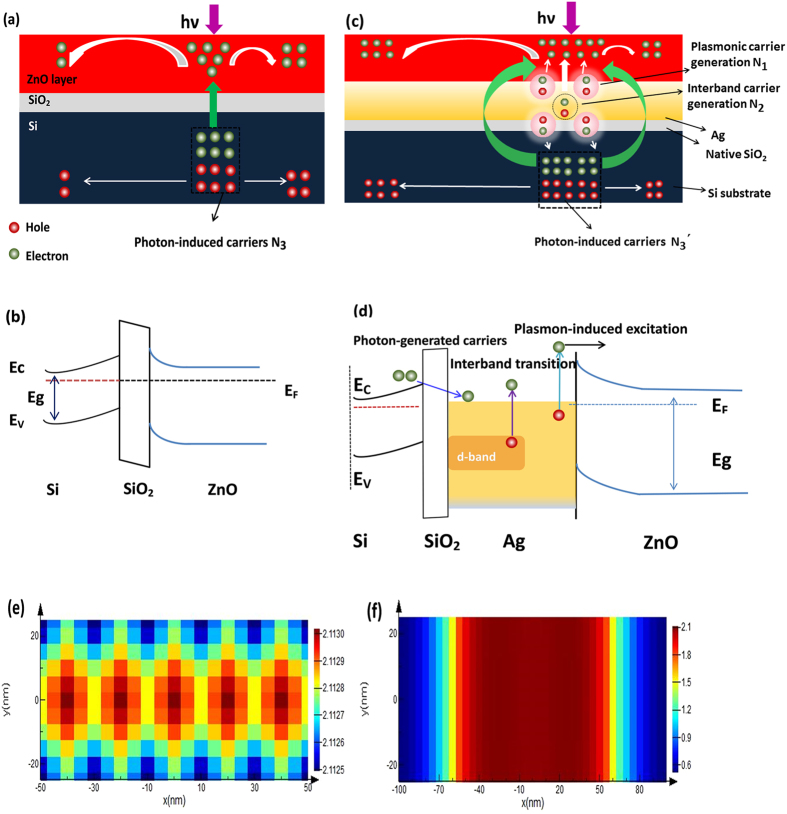
(**a**) The general schematic LPE model of the ZnO/Si nanostructure. (**b**) Energy band diagram of ZnO/Si (substrate) sample. (**c**) The schematic mechanism of the surface plasmon polariton induced excitation of interband and intraband transition as well as enhanced photon-induced carriers excitation by the interactions between the incident laser and Ag clusters on ZnO/Ag interface. (**d**) Energy band diagram of ZnO/Ag/Si samples. (**e**,**f**) The 100 nm-scale and 200 nm-scale distribution of the local field intensity simulated by FDTD in this nanostructure.

It has been well accepted that the metallic LSPRs can trigger both plasmonic excitation and interband excitation^27^. Besides, since the samples in this report were fabricated on the Si substrate, the silver associated LSPRs could also lead to an amplified direct photoelectron generation process owing to its large absorption cross section and high localized optical intensity^24^,^28^. Obviously, if that happens, the output of current of IV curve (under the same applied voltage and the uniform incident laser) will increase proportionally to PL intensity. This is because the greater the intensity, the more LSPRs-induced carriers will participate in conducting (see Fig. 3c). Thus, to further confirm the presence of LSPRs, we measured the IV curves of five samples under light illumination (see Fig. 2b). It can be seen that the sample 3 outputs the biggest current, and the values of output current of sample 1, sample 2 and sample 4 (including the control sample) decrease accordingly in sequence of their PL intensities. These results show that IV curves of the samples are well consistent with their PL measurements and also consistent with our foregoing analysis.

Based on these LSPRs-related results, the greatly enhanced LPE can be well interpreted. For general PN junction type or metal-oxide-semiconductor structures, taking the control sample ZnO/Si as an example, the LPE operation mechanism^8^,^29^ can be interpreted as following (please refer to Fig. 3a,b): When the incident laser impinged at one point on the surface of the structure, photons with energies larger than the energy bandgap of Si will generate electron-hole pairs inside the semiconductor substrate. After a very short while, the excited electrons will tunnel into the ZnO layer at the laser spot position through the Schottky Barrier (SB) while the holes are left in the semiconductor. Then the excess electrons in ZnO and holes in Si would generate a concentration gradient laterally between the illuminated spot and the unilluminated area due to the non-equilibrium state. Later then, the photon-generated electrons flow diffuses in an exponential way along the ZnO side from the laser spot position to the ohmic indium contacts which is taking charge of collecting the diffusing carriers and hence ultimately form the lateral photovoltaic output numerically described as the formula below^8^,^29^:


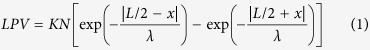


Here 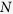 is the electrons density at the laser position 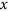 of the ZnO layer, 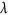 is the electron diffusion length in the nanoscale ZnO film which is approximately several millimeters according to our previous works^8^,^29^. 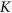 is the proportionality coefficient related to electron charge, fermi level, and the temperature. 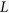 is the distance of the two contacts. Ideally, when 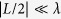, the expression of LPV and its sensitivity can be simplified as:


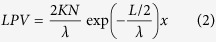



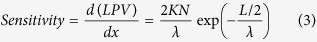


In fact, as recorded from the Fig. 1a, we have found that all of the LPV outputs had made acceptable linear changes versus the laser spot displacement, showing a relatively large electron diffusion length (Here the length of 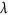 can be obtained from measuring the exponentially decreasing LPV outputs of the region outside the two collecting contacts as we have reported before^8^. In this letter, to focus our discussion on the linear changing parts and further explore their underlying mechanisms, we did not show the LPV performances (exponential curves) outside the two electrodes). Obviously, these experimental results basically agree with what the equation (2) have analyzed.

However, it is intriguing that all the silver nanostructure embedded samples presented with LPV performances several times larger than that of the control sample. Based on the aforementioned PL spectra and IV characteristics, we think that this phenomenon was ought to be associated with the metallic LSPRs triggered by Ag granular films.

It has been well known that the metallic LSPRs induced local field can effectively drive a boasted conduction electrons excitation process since its violent oscillations resonant with the applied optical field can attenuate randomly in a very short time through a nonradiative decay mechanism including the interband transition and intraband transition (plasmonic carriers generation)^16^,^27^,^30^,^31^.

Yet here on account of the fact that the Ag nanofilm was closely constructed on the semiconductor Si substrate, the LSPRs would trigger three concurrent processes and thus generate large amount of high energy carriers. That is to say: the enhanced direct photon-induced electrons (*N*_1_ + Δ*N*_1_) yielded in the Si substrate due to the abnormal light trapping (here the density of direct photoelectron in the control sample was merely amount to *N*_1_); the plasmonic carriers coming from the intraband excitation occurred nearby the metal-dielectric interfaces Δ*N*_2_ and the interband excitation mainly due to the d-band transitions inside the silver layer Δ*N*_3_. These are all clearly illustrated in the Fig. 3c,d^ ^27,30.

As we have known that the optical absorption of the silver granules was strongly localized here, hence the excited electron quantity 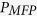 of an active silver granule that participated in the effective transfer should be figured up by integrating the product of frequency 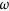, local electric field strength 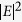 and the imaginary part of the dielectric function 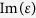 over the volume 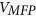 adjacent to the silver granules within the mean-free path (MFP), eventually it would come out to be^27^:


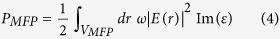


Based on this mechanism, electron density 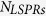 which is closely related to the 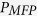 at the laser position 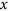 of the ZnO layer should be expressed as:


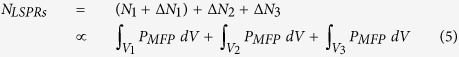


Here 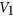, 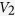 and 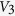 represent the valid unit volume account for the Δ*N*_1_, Δ*N*_2_ and Δ*N*_3_ respectively. Notably, the 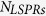 participated in the consequent formation of the LPV output is believed to be much larger than that generated in the control sample which barely valued as 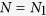.

Thus according to the equation (2) and equation (3), it can be eventually demonstrated that these high energy charge carriers 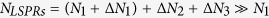 would significantly facilitate the tunneling efficiency of the excited carriers and greatly increase the quantity of diffusing electrons located at the laser spot, causing an enhanced LPV.

We want to stress here that, once again according to the equation (2), the LPV can also be regulated by changing the diffusing length 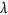 in the ZnO layer even if there are not any LSPRs effect. It is sure that the nanoscale Ag embedded interfaces could bring in a decreased resistivity 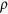, an enhanced Fermi level 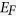 and a prolonged life-time 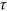 of the non-equilibrium electrons in the structures, thus eventually leading to a slightly increased. diffusing length., which can be written as:^8^,^30^


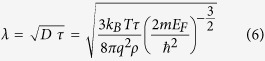


Furthermore, on the basis of our experimental data, the length of 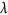 had presented with a little bit increase much less than 1 mm^8^, consistent with what the equation (6) has demonstrated. Thus these very slight changes of 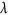 are not enough to arouse such a huge LPV enhancement in this report. Besides, according to our previous researches on the ZnO/Si nanostructures with different 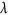
29,32, even the optimum 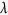 can never bring forth a LPE improvement of several folds. Thus, we believe that the large extra production of high energy conducting electrons triggered by the metallic LSPRs are meant to be the major factors of this anomalous giant LPV performance.

In addition, the rapid oscillation of the local field also produced another kind of plasmonic energy dissipation called Joule heating effect^33^, which was meant to favor the lateral diffusion process inside the ZnO layer owing to an inherent thermalization method, i.e. through the internal electron-electron scattering, electron-phonon scattering and electron surface scattering as well^33^,^34^,^35^.

To gain further insights into this metallic LSPRs effect, the spatial fluctuations of the resonant electromagnetic field intensity for the silver nanoscale granules are simulated with the finite-difference time-domain (FDTD) method under the setting periodic boundary conditions^36^. As shown in the Fig. 3e,f, the left-hand image demonstrate the intense spatial localization and remarkable field enhancement of the Ag donated surface plasma strength confined on a nanoscale 100 nm*40 nm x-y plane while the right-hand graphic page unequivocally reflect the evanescent waves nature of this localized resonant field in a subwavelength region, where the various relucent colors represent a set of field intensity dissipated from high to low. These results are well fitted with the intrinsic characteristics of the LSPRs as we have discussed above.

In summary, we have discovered a LSPRs-based giant lateral photovoltaic effect (LPE) in ZnO/Ag/Si structures for the first time. The new operation mechanism behind the LSPRs constitutes a sharp contrast to the traditional LPE mechanism and can be expected to improve the LPV performance at a rate of several times with a simple preparation process and a relatively low cost, suggesting a brand new LSPRs approach for tailoring LPE-based devices. We believe it will also open avenues of research within current photoelectric sensors area.

## Additional Information

**How to cite this article**: Zhang, K. *et al.* Localized surface plasmon resonances dominated giant lateral photovoltaic effect observed in ZnO/Ag/Si nanostructure. *Sci. Rep.* **6**, 22906; doi: 10.1038/srep22906 (2016).
